# Extracorporeal membrane oxygenation support in oncological thoracic surgery

**DOI:** 10.3389/fonc.2022.1005929

**Published:** 2022-11-25

**Authors:** Giuseppe Mangiameli, Alberto Testori, Ugo Cioffi, Marco Alloisio, Umberto Cariboni

**Affiliations:** ^1^ Division of Thoracic Surgery, IRCCS Humanitas Research Hospital, Rozzano, Milan, Italy; ^2^ Department of Biomedical Sciences, Humanitas University, Milan, Italy; ^3^ Department of Surgery, University of Milan, Milan, Italy

**Keywords:** ECMO, thoracic surgery, oncological surgery, lung cancer, NSCLC

## Abstract

The use of extracorporeal lung support (ECLS) during thoracic surgery is a recent concept that has been gaining increasing approval. Firstly introduced for lung transplantation, this technique is now increasingly adopted also in oncological thoracic surgical procedures. In this review, we focus on the cutting-edge application of extracorporeal membrane oxygenation (ECMO) during oncological thoracic surgery. Therefore, we report the most common surgical procedures in oncological thoracic surgery that can benefit from the use of ECMO. They will be classified and discussed according to the aim of ECMO application. In particular, the use of ECMO is usually limited to certain lung surgery procedures that can be resumed such as in procedures in which an adequate ventilation is not possible such as in single lung patients, procedures where conventional ventilation can cause conflict with the surgical field such as tracheal or carinal surgery, and conventional procedures requiring both ventilators and hemodynamic support. So far, all available evidence comes from centers with large experience in ECMO and major thoracic surgery procedures.

## Introduction

**Figure 1:**
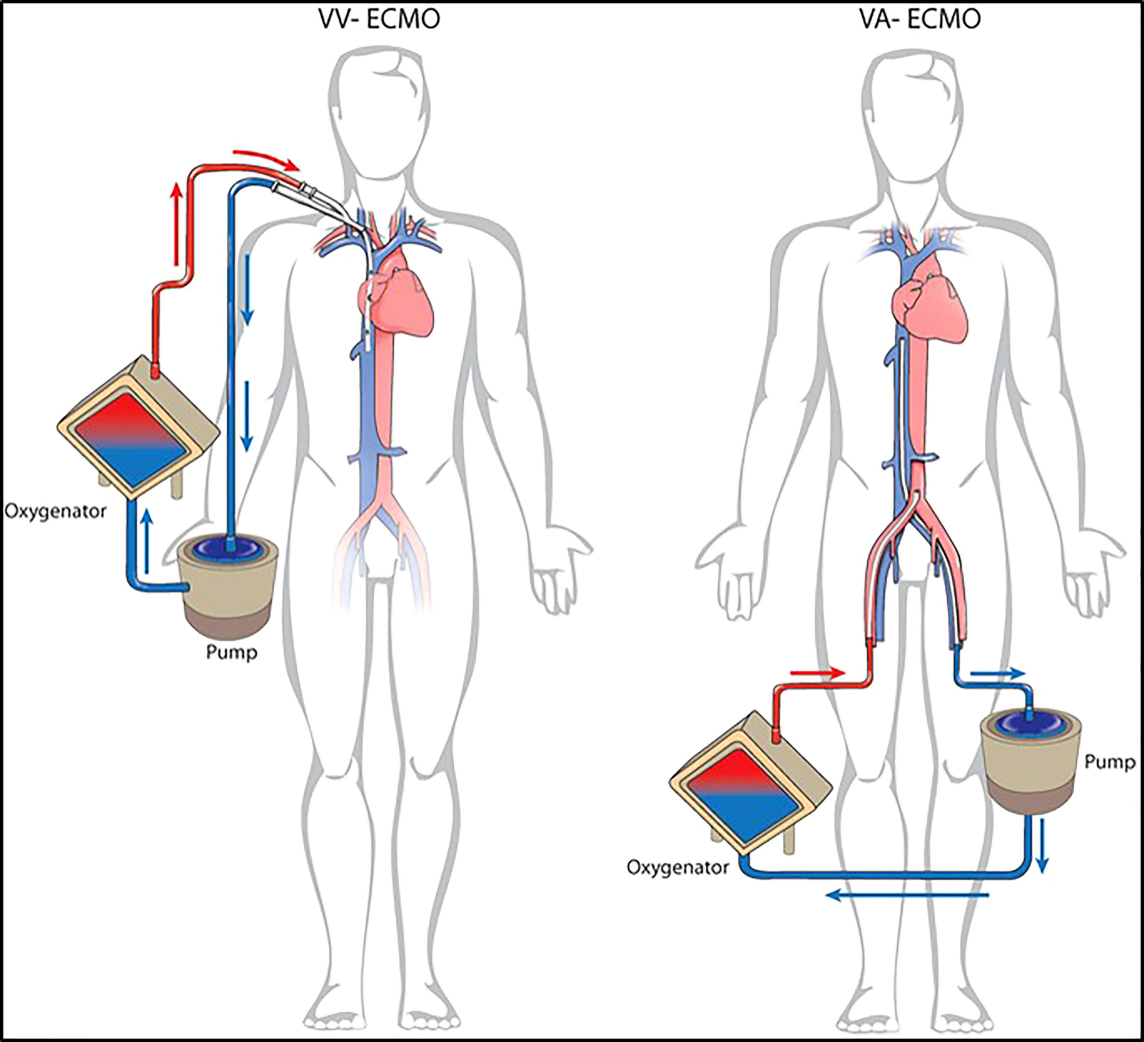
Different configuration for ECLS. V-V ECMO and V-A ECMO.

The use of extracorporeal lung support (ECLS) during thoracic surgery is a recent concept that has been gaining increasing approval. Extracorporeal membrane oxygenation (ECMO) is a mechanical ECLS normally adopted in surgery to remove CO_2_, oxygenate, or provide hemodynamic support or a combination thereof during demanding cardiovascular procedures (1). In particular, ECMO involves the use of a centrifugal pump to drive blood from the patient through an externalized membrane oxygenator system for carbon dioxide and oxygen exchange before returning to the patient. Two different forms of ECMO are actually available: veno-venous (V-V) and veno-arterial (V-A) (see **Figure 1**).

Veno-venous (V-V) ECMO is the most common ECLS system adopted in thoracic surgery.

Indeed, V-V ECMO is used in severe and refractory adult respiratory failure and requires only peripherally placed venous catheters. Blood is drained from and reinfused into central veins. Thus, V-V ECMO allows excellent oxygenation of vital organs by an inflow directed to the right atrium.

Veno-arterial (V-A) ECMO is used for hemodynamic support with or without respiratory failure because, in addition to assisting in gas exchange, it can increase cardiac output. In this configuration, blood is drained from the venous side and reinfused in the arterial system to provide hemodynamic support. The quality of oxygenation to the vital organs depends on the insertion site of the inflow cannula: (i) low in case of peripheral V-A ECMO and (ii) optimal in case of central V-A ECMO. According to specific indications, these ECLS assistances can be introduced peripherally or centrally (2) by using a minimal amount of heparinization compared to CPB (3).

ECMO was initially introduced in the field of thoracic surgery thanks to lung transplantation (4), during which more thoracic surgeons gained increasing experience allowing widespread use in the oncological field as well (5). The spread of ECMO in oncological surgery has also been justified by the fact that the ECMO systems have a significantly lower impact than the traditional cardiopulmonary bypass (CPB) in producing transient immunosuppression, preventing the spread or growth of hidden malignant cells (6). Furthermore, according to several authors, tumor cells contaminated in the CPB reservoir blood might spread through the arterial cannula, representing a risk for tumor dissemination (6, 7).

To date, the use of ECMO is usually limited to some oncological lung surgery procedures exclusively requiring an adequate ventilation support or associated with a hemodynamic support. Thus, only few data are reported in literature suggesting a favorable result for ECMO in general nontransplant thoracic surgery.

In this article, we briefly report an overview about the state of art of the application of ECMO in oncological thoracic surgery. In particular, we report the most common surgical procedures in oncological thoracic surgery that can benefit from the use of ECMO. They will be classified and discussed according to the aim of ECMO application.

## Procedures requiring an adequate ventilation

In this setting, the use of ECMO is usually limited to certain lung surgery procedures where adequate ventilation is not otherwise feasible. In these cases, V-V ECMO is the most common procedure used because a cardiovascular support is not mandatory (8). Based on the clinical status and the medical history of surgical candidates, two common scenarios are usually possible.

### Surgery in patients with a history of previous extensive contralateral pulmonary resection including pneumonectomy

In this scenario, thoracic surgery is usually performed by using short intermittent apneic phases; thus, multiple atypical lung resections or a planned anatomical resection with radical lymph node dissection could be challenging due to the limited surgical exposure.

To date, several case reports have confirmed that V-V ECMO is a suitable ECLS technique for improving hematosis during surgery when performing lung resections in one-lung patients after pneumonectomy compared to severe respiratory failure patients with problematic one-lung ventilation (3, 8).

Redwan et al. reported one of the most important experiences with the intraoperative use of ECLS; in particular, he reports the adoption of different veno-venous low-flow and high-flow modes adapted to the individual patient requirements. There are two possible scenarios (9).

The first one involves patients who have previously undergone pneumonectomy. In these patients, performing an anatomical resection with radical lymph node dissection could be a challenge. In a no- ECMO setting, these patients usually undergo surgery with short intermittent apneic phases, and the subsequent limited surgical exposure might affect the oncological accuracy. Interestingly, Redwan et al. have reported the use of apneic phases up to 45 min under low-flow V-V ECLS in combination with apneic oxygenation when performing anatomical segmentectomy with radical lymph node dissection in three patients affected by NSCLC and history of previous pneumonectomy (two left sided and one right sided) (9).

The second scenario involves patients scheduled for a planned extensive surgery to the nonoperated lung with a history of previous extensive contralateral lung resection leading to an impairment of lung function and a decline in alveolar gas exchange surface. In these cases, a conventional single-lung ventilation during the entire surgical time may be inadequate and an intermittent double-lung ventilation is needed to maintain sufficient gas exchange. The practical surgical problem due to intermittent ventilation is the consequent reinflating of the lung that interferes with an optimal atelectatic state, which is essential to perform accurate oncological resection especially for multi-lobar metastasectomy. Therefore, in such cases, the German group reports the adoption of a high-flow V-V ECLS that allowed safe and extensive metastasectomy under optimal lung atelectasis in a patient affected by adenoid cystic carcinoma of the mandibular gland with bilateral pulmonary metastases and a history of previous extensive contralateral thoracic surgery (left lower lobe lobectomy and triple-wedge resections of the left upper lobe). He presented with multiple right-sided metastases in all three lobes. In this case, an extensive metastasectomy was performed including multiple atypical lung resections and two anatomical segmentectomies, under optimal lung atelectasis without any respiratory impairment (9).

### Surgery in patients having severely compromised pulmonary function

In patients having severely compromised pulmonary function, conventional single-lung ventilation may be problematic mainly due to hyperinflation and bronchial obstruction. In chronically pathological lungs, the intraoperative high-pressure ventilation could cause additional trauma, leading to complications such as secondary induced pneumothorax, prolonged air leakage, or barotrauma.

The use of ECMO for supporting a compromised pulmonary function during surgery was first reported in nononcological cases. For example, the use of V-V ECMO has been reported in cases of ARDS or respiratory failure allowing, under single-lung ventilation, limited lung resection (atypical resection or segmentectomy) for nononcological diseases (aspergillosis or lung abscess) (10, 11).

In the oncological field, the application of ECMO is rare. The first useful and safe adoption of a V-V ECLS in performing an oncological lung resection in a patient having a severe compromised lung function was reported by Redwan et al. (12).

In this scenario, the strategy adopted was the placement of a single-site cannulation low-flow V-V ECMO providing a sufficient intraoperative support assuring “ protective” single-lung ventilation and avoiding additional barotrauma, which usually is a consequence of a high -pressure single ventilation of a pathological lung. Furthermore, by adopting the strategy of a single-site venous cannulation, all the described possible complications due to the arterial cannulation were avoided.

In particular, they reported a challenging surgical procedure performed in a 75-year-old male patient with a long-standing history of chronic obstructive pulmonary disease (Gold stage IV) and severe bullous emphysema. The surgery was a right lower lobe lobectomy and en bloc S6 segmentectomy, but due to tumor central localization with invasion of the lateral wall of the bronchus intermedius and pulmonary artery, bronchial and right pulmonary artery sleeve resection with reimplantation of the middle lobe bronchus was necessary. The entire procedure was performed under uncomplicated single-lung ventilation thanks to the low-flow V-V ECMO support through a double-lumen twin-port cannula. Bronchial and vascular anastomoses were performed under an apnea phase of 30 min to enable optimal surgical exposure without any respiratory or hemodynamic changes. At the end of the procedure, the patient was normally extubated in the operating room and ECLS was successfully removed. The postoperative course was uneventful and the patient was discharged on the 16 th postoperative day but needed postsurgical intensive respiratory therapy.

The same strategy was adopted by Redwan et al. to perform a VATS right upper lobectomy in a 69- year-old woman having a squamous cellular carcinoma of the right upper lobe (cT2N0) and 33% of predicted FEV1 (9).

Logically, this type of ECMO indication for supporting patients with severe respiratory disease is limited in the oncological field because ECMO allows one to safely perform surgery but the preoperative severe respiratory status is not reversible and usually worsened by surgery.

All cited studies are reported in **Table 1**.

**Table 1:** Studies reporting the use of ECMO in oncological procedures requiring an adequate ventilation.

Author	Year	No. of pts	Indications	Lung resection	ECMO type	Rationale
Redwan (9, 12)	2015	3	NSCLC	Segmentectomy (IIr, IIIr, VIIIl)	V-V low flow	Previous pneumectomy
		2	NSCLC	Extended RLL with right PA sleeve resection, reimplantation of the ML bronchus and en bloc segmentectomy (II) RUL lobectomy	V-V low flow	Hypercapnia massive emphysema
		2	Lung metastases	Extensive metastasectomy Multiple wedge resection of the LUL	V-V high flow	Previous extensive metastasectomy of the left lung Previous left-sided single-lung transplantation due to end-stage fibrosis with nonfunctioning right fibrotic lung

NSCLC, non-small cell lung cancer; r, right; l, left; V-V, veno-venous; RLL, right lower lobectomy; ML, middle lobe; RUL, right upper lobectomy; LUL, left upper lobectomy; PA, pulmonary artery.

## Procedures requiring an obstacle-free surgical field

ECMO is the ECLS of choice for the treatment of T4 NSCLC presenting with carinal extension requiring complex tracheobronchial reconstruction. The main limitation of conventional ventilation during complex tracheobronchial reconstruction is the presence of disturbing lines or tubes that obstruct the operative field. In these circumstances, hemodynamic stability or cardioplegia is not necessary and a good oxygenation in addition to removal of CO_2_ and a complete ventilator support could entirely be assured by ECMO (13). According to the type of tracheobronchial resection and the need to extend or not the surgical resection to the descending aorta or atrium, the use of both V-V and V-A ECMO has been reported in literature.

Initially, sporadic case reports and small cohort studies have reported the successful use of ECLS for tracheal surgical or endoscopic procedures (14, 15). One of the first and larger shared experiences in this field of ECMO application is reported by Lang et al. This Austrian surgical group has reported their experience with intraoperative V-A ECMO in performing complex tracheobronchial resection procedures in 10 patients with thoracic malignancies with excellent results in terms of mortality (0%) and R0 resection rate (89%) (16, 17).

In 2021, Koryllos et al. reported a series of 24 patients undergoing combined complex lung, carinal, aortal, or left atrial resections for oncological reasons by using intraoperative ECMO (16). They performed eight carinal resections, reporting a 78% complete resection (R0) rate and a 25% 30-day mortality. The authors report that the use of V-V ECMO for total respiratory support enabled an excellent surgical field exposure without any required interruption for mechanical ventilation. Furthermore, this strategy allows the reduction of intraoperative ventilation trauma in a group of patients with a high risk of postoperative ARDS, which is a common complication previously reported in these complex tracheobronchial procedures (17, 18). Finally, none of the patients required a V-A cannulation for additional circulatory support.

Recently, Spaggiari et al. have reported their preliminary results of ECMO-assisted tracheal sleeve pneumonectomy (TSP) for cancer in six patients (19). It is a significant experience considering that all the procedures were performed in an oncological setting and, in the last 10 years, only three studies have reported ECMO-assisted TSP for lung cancer, with only three patients described (12, 17, 20).

TSP for treating lung cancer is an old procedure described by Abbot in 1950 (21); this technique is reserved for exceptional cases presenting tracheal carina involvement. This operation is extremely challenging for thoracic surgeons, anesthesiologists, and pulmonologists because of intra- and postoperative management. Several intraoperative strategies have been described to assure a correct ventilation during this type of surgery in these patients, each of these presenting specific limitations.

The cross-field ventilation through a classical endotracheal tube has some major difficulties such as the closure of the left upper bronchus due to its different anatomical length in the patients, the continuous tube dislocation, the possible blood lung aspiration during the dislocation of the tube, and the ischemic damage of the proximal end of the main bronchus due to balloon overinflating.

Similarly, other proposed ventilation strategies, such as intermitted cross-field ventilation, “apneic oxygenation”, or jet ventilation, have some limitations, such as intraoperative hypercapnia, the occurrence of lung atelectasis, which can facilitate postoperative infective complications, or possible submucosal endobronchial cancer dissemination (19, 22).

As reported by Spaggiari et al., ECMO-assisted surgery assures adequate respiratory support, hemodynamic stability, an improved brain and myocardial oxygenation, and a lower risk of bleeding complications with a “clean” surgical field without cross-field tubes.

During the time of ECMO activation, the use of modern heparin-coated vascular cannulas prevents episodes of deep venous thrombosis and, additionally, they can be maintained in the case of postoperative instability or if needed. Finally, the theoretical risk of tumor cell spread during ECMO is negligible, considering the absence of the cardiotomy reservoir and the fact that ECMO is a closed circulatory system starting only after significant vessel ligation and lung removal. In their reported experience, they did not observe cannula- related complications or complications during ECMO assistance. The mean duration of assistance was short (38 min); it did not require excessive anticoagulation, and the rapid normalization of coagulation after ECMO use avoided any risk of bleeding. According to the authors, the use of ECMO during carina resection and tracheobronchial reconstruction improved surgical results. The reported advantages of this strategy are the following: the anastomosis can be completed efficiently; technical errors that could be fatal in the postoperative period can be avoided; the lack of left main bronchus manipulation by the endotracheal tube reduces ischemic damage of the stump, probably reducing the risk of dehiscence; the lack of contralateral lung atelectasis due to ventilation overpressure in the cross-field ventilation; and the inevitable passage of blood within the left main bronchus during the intervention due to the continuous manipulation of the bronchus for ventilation.

Recently, Martinod has reported the long-term follow-up and results of his series of 35 adult patients subjected to airway replacement using stented aortic matrices. In this series, 29 patients (82.9%) were operated for malignant lesions, and the use of V-V ECMO was reported in 4 (11.4%) out of 35 patients (23). All cited studies are reported in **Table 2**.

**Table 2:** Studies reporting the use of ECMO in oncological procedures requiring an obstacle-free surgical field or both ventilatory and hemodynamic support.

Author	Year	No. of pts	Indications	Lung resection	ECMO type	Rationale
Lang (17)	2015	10	NSCLC: 7 Carcinoid:2 Adenoid cystic carcinoma: 1	Complex bronco-tracheal reconstructions	V-A	Avoiding cross-field or jet ventilation
Redwan (9)	2015	1	NSCLC	Left-sided pneumonectomy with carinal sleeve resection	V-V	Avoiding cross-field or jet ventilation to the right lung
Koryllos (24)	2021	8	NSCLC	Complex bronco-tracheal reconstructions	V-V	Avoiding cross-field or jet ventilation to the right lung
Spaggiari (19)	2021	6	NSCLC	Left-sided pneumonectomy with carinal sleeve resection	V-V	Avoiding cross-field or jet ventilation to the right lung
Costantino (20)	2019	1	NSCLC	Left-sided pneumonectomy with carinal sleeve resection	V-A	Avoiding cross-field or jet ventilation to the right lung
Mazzella (22)	2021	1	NSCLC	Right-sided pneumonectomy with carinal sleeve resection	V-V	Avoiding cross-field or jet ventilation to the right lung
Martinod (23)	2022	4	Non reported	Airway replacement using stented aortic matrices	V-V	Avoiding cross-field or jet ventilation
Lang (17)	2015	3	NSCLC: 2 Sarcoma: 1	Descending aorta Inferior vena cava	V-A	Hemodynamic support
Koryllos (24)	2021	6 9	NSCLC: 6 NSCLC:7 Sarcoma: 2	Descending aorta Left atrium Left atrium	V-A	Hemodynamic support
Novellis (25)	2022	1	NSCLC	ULL with pulmonary artery angioplasty.	V-A	Hemodynamic support

NSCLC, non-small cell lung cancer; V-A, veno-arterial; V-V, veno-venous; ULL, upper left lobectomy.

## Procedures requiring both ventilatory and hemodynamic support

As previously mentioned, V-A ECMO is the ECLS of choice for procedures that require both ventilatory and hemodynamic support. Usually, a very limited number of reports have considered the use of V-A ECMO in patients undergoing non-cardiac surgery (20, 26).

Two different scenarios are usually reported in literature for this type of ECMO indication.

The first one involves T4 NSCLC patients or patients affected by large sarcoma who need a simple lung resection or complex tracheobronchial reconstructions associated with the resection of the great vessels of the left atrium. Usually, surgery for centrally located cancers with wide infiltration of the left atrium or the descending aorta is related to challenging intraoperative conditions, making the adoption of an ECLS attractive for thoracic surgeons (27).

In this case, according to Klepetko’s experience, V-A ECMO should be considered a safe alternative to CPB, avoiding its disadvantage when performing this type of extended surgery. Notably, in 2011, Klepetko et al. reported a series of nine cases of thoracic malignancies: in three of them, V-A ECMO was used to perform two descending aorta resections (in two patients affected by NSCLC) and one inferior vena cava resection (in one patient affected by synovial sarcoma) in addition to lung surgery. Based on their experience, the authors recommend that ECMO should also be used in performing resection of great vessels, with the traditional CPB support reserved for open resection of either the left or the right atrium, resection of the aortic arch, or central resection of the pulmonary trunk (16).

Similarly, Koryllos et al. in their series of 24 patients reported the use of V-A ECMO in two particular patient groups. The first involved resections of the left lung and the descending aorta (*n* = 7) and the second involved resections of the lung and left atrium (*n* = 9).

In the first group of patients, a V-A ECMO was the chosen ECLS support in estimating challenging cases with a setting for partial circulatory support (50% of the cardiac output) during longer periods of aortic clamping. Otherwise, in cases of left atrial tumor infiltration, a VV-A- ECMO setting for total circulatory support was the adopted strategy during surgery (24).

The second scenario involved patients affected by early-stage lung cancer with severe heart failure who would be excluded from surgery (the standard treatment) because of prohibitive perioperative risk (27–29). In these cases, ECMO could be a suitable option by assuring both circulatory and respiratory support.

Recently, Novellis et al. reported the use of V-A ECMO as a tool to provide temporary cardiac support in a patient with severe impaired ejection fraction (EF) affected by resectable lung cancer and described the benefits of this new method (25). In particular, they reported the intraoperative fast-track use of V-A ECMO in a stage cIIA lung cancer patient with arterial infiltration and severe postischemic dilated cardiomyopathy (EF: 23%) subjected to a left upper lobectomy with angioplasty of pulmonary artery (25). Immediately after surgery, the circuit was removed, the heparin reversal was not administered, and the patient was extubated 3 h later in the intensive care unit. Finally, the patient was discharged in good general condition after an uneventful postoperative course (25).

According to the authors, when hemodynamic support is the main indication for the use of an ECLS device, V-A ECMO offers the most favorable profile because it completely supports the hemodynamic and respiratory function. In their experience, this fast- track strategy allowed the mitigation of all of the complications (bleeding, infection, thrombosis, and ischemia) usually associated with prolonged V-A ECMO support. The use of fast- track V-A ECMO enables fragile patients to be supported during the most challenging phase of the entire perioperative course. In fact, because hypotension, hypercapnia, hypoxia, pulmonary hypertension, tachycardia, and bleeding are more commonly observed intraoperatively than postoperatively, it is reasonable that the hemodynamic support should be maximal intraoperatively. Nevertheless, the cardiac risk in a patient with severe impaired EF remains higher than in a healthier population and can be mitigated only with meticulous postoperative monitoring. All cited studies are reported in **Table 2**.

## Conclusion

The concept of ECMO-assisted noncardiac oncological thoracic procedures is becoming increasingly attractive. Emerging evidence supports the use of ECMO as both respiratory and circulatory support to facilitate stable intraoperative conditions for satisfying the main goal of oncological R0 resection in case of complex tracheobronchial, atrial, or combined lung-aortic resections and to allow surgery in patients with respiratory limitations or cardiological comorbidities. In these latter cases (impaired lung and/or cardiac function), ECLS allows a safe surgical resection, but it cannot reverse baseline clinical conditions of the patients and modify their high postoperative mortality rate. To date, all the available evidence comes from centers with large experience in ECMO and major thoracic surgery procedures.

## Author contributions

GM and UCa contributed to conception and design of the study. GM wrote the first draft of the manuscript. All authors contributed to manuscript revision, read, and approved the submitted version.

## Acknowledgments

We thank Dr. Gerardo Cioffi, native speaker, for reviewing the English language.

## Conflict of interest

The authors declare that the research was conducted in the absence of any commercial or financial relationships that could be construed as a potential conflict of interest.

The reviewer FR declared a past co-authorship with the author UCi to the handling editor.

## Publisher’s note

All claims expressed in this article are solely those of the authors and do not necessarily represent those of their affiliated organizations, or those of the publisher, the editors and the reviewers. Any product that may be evaluated in this article, or claim that may be made by its manufacturer, is not guaranteed or endorsed by the publisher.
